# The Enhanced Interactive Physical and Cognitive Exercise System (iPACES^TM^ v2.0): Pilot Clinical Trial of an In-Home iPad-Based Neuro-Exergame for Mild Cognitive Impairment (MCI)

**DOI:** 10.3390/jcm7090249

**Published:** 2018-08-30

**Authors:** Kathryn Wall, Jessica Stark, Alexa Schillaci, Emilie T. Saulnier, Elizabeth McLaren, Kristina Striegnitz, Brian D. Cohen, Paul J. Arciero, Arthur F. Kramer, Cay Anderson-Hanley

**Affiliations:** 1 Healthy Aging & Neuropsychology Lab, Union College, Schenectady, NY 12308, USA; wallk@union.edu (K.W.); jhs55@miami.med.edu (J.S.); aschillaci15@aol.com (A.S.); 2 Biology Department, Union College, Schenectady, NY 12308, USA; cohenb@union.edu; 3 1st Playable, Troy, NY 12108, USA; tobi@1stplayable.com (E.T.S.); elizabeth@1stplayable.com (E.M.); 4 Computer Science Department & Neuroscience Program, Union College, Schenectady, NY 12308, USA; striegnk@union.edu; 5 Health & Human Physiological Sciences Department, Skidmore College, Saratoga Springs, NY 12866, USA; parciero@skidmore.edu; 6 Center for Cognitive & Brain Health, Psychology Department, Northeastern University, Boston, NY 02115, USA; a.kramer@northeastern.edu

**Keywords:** exercise, exergame, mild cognitive impairment, neurocognitive disorder, dementia, Alzheimer’s, executive function, IGF-1, cortisol, older adult

## Abstract

Given increasing longevity worldwide, older adults and caregivers are seeking ways to curb cognitive decline especially for those with mild cognitive impairment (MCI, now mild neurocognitive disorder, mNCD, Diagnostic and Statistical Manual of Mental Disorders, 5th ed. (DSM-V). This quasi-experimental, within-subjects pilot clinical trial was designed to replicate and extend the study of cognitive benefits for MCI by improving upon our prior interactive Physical and Cognitive Exercise Study (iPACES^TM^ v1.0) by increasing the usability of the neuro-exergame and exploring possible underlying neurobiological mechanisms. Older adults were enrolled in a three-month, in-home trial of a portable neuro-exergame (iPACES™ v2.0) where participants pedaled and steered along a virtual bike path (Memory Lane™). Neuropsychological function was assessed at baseline after component familiarization intervals (e.g., two weeks of exercise-only, game-only, etc.) and after three months of interactive neuro-exergame intervention. Fourteen participants were enrolled in the study and seven completed the final evaluation. Intent-to-treat analyses were conducted with imputed missing data (total *n* = 14). Significant improvement in executive function (Stroop) was found (*d* = 0.68, *p* = 0.02) only. Changes in salivary biomarkers (cortisol and insulin-like growth factor 1; IGF-1) were significantly associated with improved cognition. Further research is needed, but pilot data suggest that a portable in-home neuro-exergame may be an additional, practical tool to fight back against cognitive decline and dementia.

## 1. Introduction

Aging may be accompanied by impactful cognitive changes such as executive function decline, which is often seen in Alzheimer’s disease and related dementias (ADRDs) [1]. There has been growing concern worldwide regarding the increasing prevalence of cognitive decline in our aging population [2]. Dementia (DSM-IV) [3], which is now a major neurocognitive disorder (MND, DSM-V) [4], has been an umbrella term for the general loss of cognitive functions. This often includes impaired performance in domains of intelligence, memory, language, visuo-spatial, and/or executive function. Dementia may also include significant changes in personality or activities of daily living [4]. A mere 15 years ago, 3.8 million older adults (65+) in the United States were diagnosed with dementia [5]. In the United States alone, dementia cases have nearly doubled, reaching more than five million cases [6] and it is projected that worldwide incidence will surpass 115 million by 2050 [7]. Since ADRDs are often marked by declines in executive function that often lead to a loss of independence [8]. Research strives to identify preventative measures to delay the onset of or ameliorate cognitive decline often first presenting as mild cognitive impairment (MCI; DSM-IV [3]). Exercise has shown promising results in slowing the decline in cognition [9]. However, many older adults do not engage in adequate exercise [10]. The present pilot study was concerned with replicating and extending a prior finding than a more engaging neuro-exergame in which physical and mental exercise were intertwined interactively could benefit cognitive performance, specifically executive functioning [11], and also exploring whether changes in biomarkers might correspond with improvements.

### 1.1. Physical Exercise and Aging

Recent and prior meta-analytic reviews [9,12,13] have found that numerous well-controlled studies have strengthened the claim that physical exercise can positively impact cognitive functioning in later life. Reviews continue to call for more well-controlled clinical trials to incorporate innovations that will reach and engage pre-clinical cases such as MCI where cognitive decline might go undetected yet harbor underlying neuropathology that could potentially be ameliorated with intervention [14,15].

### 1.2. Physical Exercise and Cognitive Decline (MCI)

A number of meta-analytic reviews have explored the effect of physical exercise on cognition specifically in the MCI population [16,17,18]. Results revealed that physical exercise was positively associated with global cognition. Of all the types of physical exercise interventions, aerobic exercise consistently had a medium effect size on global cognition in the MCI population [17] and an executive function in older adults [19]. Recent work assessing older adult participants also revealed that a physical weakness was associated with an increased amount of amyloid beta in the brain, which is an indicator of Alzheimer’s disease (AD) [20]. Physical exercise and its effect on cognition in the AD population specifically has indicated that physical exercise is among the most impactful of interventions for improving cognition [21].

Despite the preponderance of research supporting physical exercise as a useful intervention in cognitive aging and MCI, most older adults do not get the recommended amount of exercise each week [10]. Research from our own lab chose exergaming as a way to motivate increased exercise compliance and adequate dosing with the intent of maximizing cognitive benefit [22]. We found that older adults pedaling along a virtual reality pathway on a stationary bike (aka “cybercycle”) accrued greater cognitive benefit after three months than those who pedaled a traditional stationary bike. Yet, in the end, motivation and the dose were not the differentiating factors. Instead it appeared that the combination of physical and mental exercise yielded additive and synergistic effects [22]. Recent research has also shown that other technological motivations such as smart watches may encourage exercise [23]. Animal research has also contributed to our understanding by revealing that physical and mental exercise have different neurobiological impacts. Physical exercise leads to neuronal proliferation and mental exercise (aka “environmental enrichment”) leads to neuronal survival [24,25,26]. Similarly, human research has reported that physical and mental exercise are associated with different structural and functional differences in the brain [27]. This research aimed to maximize a cognitive benefit by combining interactive cognitive and physical exercise (herein iPACES™), and tried to identify markers of underlying neurobiological mechanisms.

### 1.3. Mental Exercise (Cognitive Training) and Cognitive Decline (MCI)

The above finding from our initial cybercycle study, in retrospect, is perhaps not surprising given that there is a considerable growing, albeit controversial, literature (including meta-analyses of controlled trial) indicating the effects of cognitive training interventions [28,29,30,31,32]. Studies that have employed cognitive interventions alone such as computerized tasks, have been suggested to improve cognitive performance in older adults [29,33,34,35]. However, many of these studies did not address cognitively declining samples [36,37] and critiques of the literature question the transfer of apparent cognitive gains [38,39,40,41]. Yet, given larger effect sizes and fewer side effects than medications, the American Academy of Neurology (AAN) [42] and others recommend that health care practitioners working with patients with MCI advocate for physical and mental exercise over and above pharmacologic interventions [21,42,43,44,45].

### 1.4. Combined Physical and Mental Exercise for MCI (e.g., Combined/Tandem or Interactive/Neuro-Exergaming)

Furthermore, research in humans suggests that neuroplasticity is induced by exercise [46,47], which may prime the substrate prior to or in concert with cognitive exercise and reviews of published studies have found that, when physical exercise is combined with mental exercise, there are added cognitive benefits [47,48,49,50,51,52,53,54,55]. There are many different ways to combine mental and physical exercise, including: 1. sequentially (e.g., cognitive training follows physical activity dis-synchronously often in tandem); 2. simultaneously (e.g., cognitive tasks are presented at the same time while doing physical activity, but they are “disparate,” without interactivity, as in “dual-task” paradigms); and 3. interactively (e.g., physical and cognitive activities are interwoven such that performance in one realm affects the other and vice versa) [11]. Determining which way to combine mental and physical exercise is most effective or under what participant and environmental circumstances requires additional research.

To evaluate the potential to maximize the cognitive benefit of an interactive physical exercise, we compared the impact of different levels of mental challenges in a recently completed randomized clinical trial (RCT, Aerobic and Cognitive Exercise Study [ACES]) for MCI [56]. In the ACES trial, similar outcomes were achieved by six months for participants of either: (1) pedaling and steering along a scenic bike path (exer-tour: low mental challenge); or (2) pedaling and steering through a videogame landscape tagging dragons and coins to score points (exer-score, high mental challenge) [56]. Further comparative analyses had been planned but could not be pursued due to attrition. It was difficult for MCI participants to leave their home and the commercial grade equipment was too large and expensive to distribute in homes. As a result, our lab began developing a portable neuro-exergame for use in the home. The interactive Physical and Cognitive Exercise System (iPACES™) was found to be feasible for older adult use in a single bout study in the lab (v1.0) [57]. This system also yielded promising results in an initial pilot clinical trial of older adult use in the home for three months [11].

It has thus been proposed that both mental and physical exercise interventions together may improve or slow the decline of cognitive abilities in older adults with MCI [58] including sequential/tandem or simultaneous dual-task paradigms [59,60,61,62], yielding improvements in executive function tasks, global-visual memory, processing speed [39,63,64], and improved brain health per neuroimaging [65,66]. Nevertheless, when sequential or in tandem mental and physical exercise interventions were compared to their counterpart interventions alone, they did not produce any greater cognitive improvements than one or the other [52,59,67,68,69,70,71,72,73]. Furthermore, some research has shown that, combined with physical and mental exercise or virtual-reality exergaming interventions, may be the most useful in the early (vs. more severe) stages of cognitive decline [58]. This suggests that there might be a “sweet spot” along the continuum of decline in which MCI might be best suited to extract a benefit from combination interventions. One recent meta-analysis examined four multi-component studies that included MCI samples reporting that “separate” studies (aka sequential/tandem) were more effective than “simultaneous” interventions [74]. The latter compared dual-task vs. interactive aerobic paradigms such as in the cybercycle study [22]. We have hypothesized previously that interactive paradigms are more intuitive, which simulates real-life activities that synergistically activate evolutionarily-adapted networks (e.g., move along a path while seeking a target: destination/food/enemy), vs. forced dual-task paradigms that engage neuronal networks in a competition for resources (e.g., move along a path while being distracted to compute a mathematical problem or memorize an unrelated word list). Theoretical and neurobiological explanations for synergistic effects have been offered [51] and more innovation and research is called for [75] to explore the impact and explanations behind truly interactive physical and cognitive exercises (iPACES) especially for MCI.

Simultaneous exercise and cognitive interventions have been shown to have positive influences on cognition in older adults with a variety of exercise activities yielding similar results [11,22,75,76,77,78,79,80,81,82]. Meta-analyses comparing combined interventions to mental-only and physical-only interventions indicated that the combined intervention is more effective than the individual components [50,73,74]. Additionally, recent meta-analytic work has shown that overall combined physical and cognitive exercise interventions resulted in significantly improved cognitive performance [47]. In addition, interventions where cognitive and physical activities were occurring simultaneously and were superior to those where these tasks were performed separately [47].

Reported positive physical effects of combined physical and cognitive training have led to an increase in this facet of research such that there are now enough completed trials that they can be analyzed systemically. A couple of published reviews of the literature have explored interactive exergames and their relationship to cognitive functioning in those with neurological disability and it was found that exergaming improved executive functioning with medium-effect sizes [83,84]. Mura and colleagues [83] posited that these benefits may stem from improved decision-making and visuo-spatial perception that create an increased ability to use cognitive resources. Exergaming also had these positive effects when compared to neurologically disabled participants with no intervention [83]. Recent work has also demonstrated that supportive feedback during exergames make them more enjoyable to the user [85], which might increase compliance in these interventions. There is unfortunately a lack of research comparing simultaneous or interactive (exergaming) interventions to sequential interventions, but it is hypothesized that interactive interventions could be most potent due to potentially synergistic effects [22,75,84].

### 1.5. Exercise, Cognition, and Biomarker Indicators

Insulin-like Growth Factor 1 (IGF-1), Dehydroepiandrosterone sulfate (DHEA-S), and cortisol are three biomarkers that might potentially be associated with the changes in executive functioning that occur after physical and cognitive exercise interventions [73]. IGF-1 levels have been correlated with different levels of cognitive abilities [86]. Recent studies have shown that decreased IGF-1 is associated with decreased cognition [87,88]. IGF-1 has also been shown to increase with exercise [89,90,91]. Low levels of DHEA-S are associated with many conditions such as Alzheimer’s, schizophrenia, and HIV/AIDS, which suggests that DHEA-S has a relationship to cognition [92,93]. Healthy individuals have also displayed this connection with greater levels of DHEA-S corresponding to greater cognitive abilities [94]. Additionally, cortisol has also been linked to cognitive impairments and some neurodegenerative conditions such as Alzheimer’s and other dementias [95,96,97,98,99] and has been responsive to exercise interventions in MCI [100], which makes it another important biomarker to assess in the current study.

It was hypothesized based on prior literature that:

1. cognition, more specifically executive function, would improve over the course of the three-month neuro-exergame intervention (partial replication and extension of prior findings [11,22])
2. cognitive improvement would be correlated with salivary biomarkers:
  - cortisol would be negatively correlated per [95,96]
  - IGF-1 would be positively correlated per [88]
  - DHEA-S would be positively correlated per [91,93]
3. a component familiarization period would not exceed standard practice effects [pedal-only and game-only practice periods were included to gradually train and prepare cognitively challenged participants for the more complex interactive neuro-exergaming experience (iPACES) and also these periods were anticipated to have a dual benefit of washing out any practice effects from serial cognitive testing such that the learning curve would be similar to that of published normative data] [101].

## 2. Experimental Section

### 2.1. Participants

The iPACES^TM^ v2.0 was an IRB-approved quasi-experimental pilot clinical trial (NCT03069391). A within-subjects design was employed such that participants were incrementally exposed to and trained in the independent physical and cognitive components before using the fully interactive iPACES intervention with a dual goal of washing out practice and learning effects from repeated neuropsychological evaluations. Participants were recruited through flyers, newspaper ads, demonstrations at local retirement and community centers, and through the Union College Academy for Lifelong Learning (UCALL) program. Participants sought were age 50+, sufficient visual, auditory, physical functions to participate in testing and exercise, and no known diagnosed neurological condition (e.g., epilepsy, Parkinson’s disease, and Alzheimer’s disease). Additionally, participants had to be co-residing with a partner for safety reasons (buddy system during exercise). All participants were screened with the Impaired Decision-Making Capacity structured interview (IDMC; Veteran’s Health Administration Handbook 2007) [102] and provided informed consent (if applicable, it would have been co-signed by a surrogate or legally-authorized representative per IDMC results).

Enrolled (*n* = 14, 7 pairs) were six females and eight males. The mean age of participants was 82.8 (*SD* = 3.9), mean level of education was 16.6 years (*SD =* 2.1), mean body mass index (BMI) was 24.7 (*SD* = 3.3), average baseline cognitive status was in the MCI range (23.4, *SD* = 2.8 per the screening test for MCI: Montreal Cognitive Assessment (MoCA) < 26), and all were Caucasian (largely consistent with the catchment area of recruitment in upstate New York). Enrolled participants were co-residing pairs in which each of the partners participated in exercise and assessments. The buddy system was used to limit health risks associated with a typically sedentary older adult exercising alone in their home while also serving to remind one another to complete study activities. Two participants were not able to provide useable data on most measures due to previously existing conditions and were excluded from final analyses. (One participant had macular degeneration, but could play the game and wanted to exercise with their partner and could complete some non-visual tests such as the ADAS. The other participant had more cognitive impairment than apparent at first and could not follow instructions well enough to complete some of the test, but also wanted to continue the exercise with their partner.). Of the 14 participants enrolled, seven completed the final three-month evaluation. Of those seven, four were compliant with the recommended dose of exercising 30 to 45 min/week and 3 to 5 times/week within their ideal heart rate range (averaging a minimum of 2.5 times/week allowing for 1 to 2 weeks of vacation, illness, or equipment breakdown).

### 2.2. Procedures

**Figure 1:**
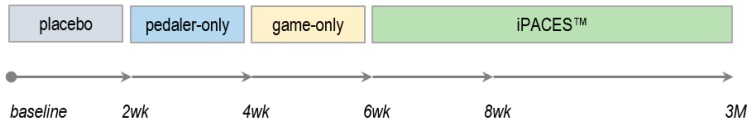
Pilot study design showing component familiarization periods leading to interactive Physical and Cognitive Exercise System (iPACES) intervention.

Once a co-residing pair expressed interest in participating in the study, they were screened to determine if they met study criteria and an in-home initial evaluation was scheduled. Two trained research assistants were present for in-home data collection so that testing of each participant could be done simultaneously in separate but adjacent parts of the home (e.g., kitchen and living room). Participants completed a battery of neuropsychological tests that focused on executive function (see measures below). Participants also provided saliva samples through passive-drool collection for the evaluation of biomarkers (per measures below). The initial evaluation also included a series of demographic, exercise history, and mood questionnaires. After each data collection point, the next relevant condition was introduced to participants (e.g., placebo, exercise-only, and game-only). For each condition, participants were asked to participate in the activities for 30 to 45 min on each occasion and 3 to 5 times per week. Evaluations were performed at weeks: 0, 2, 4, 6, 8 weeks, and after three months. An initial placebo period was used to familiarize participants with in-home evaluations (e.g., including completing cognitive tasks on a touchscreen iPad) and, during those two weeks, participants completed a set of on-screen readings on nutrition and exercise by answering a couple of simple multiple choice questions at the conclusion of each session to serve as verification. The second and third two-week windows served to introduce, familiarize, and evaluate each component of the neuro-exergame (physical exercise: pedaler-only and cognitive exercise: game-only) before introducing the interactive use of both in the full intervention (iPACES). The remaining weeks evaluated the full interactive neuro-exergame intervention of iPACES (Figure 1). In all conditions, participants were asked to record their activities in a paper log kept in a binder with study protocol instructions and other information provided at their initial evaluation. Motivation to complete the exercise was measured through a single item Likert scale. Upon study completion, participants were asked to fill out an exit interview questionnaire in which participants described how enjoyable the exergame intervention was and where the exergame intervention could be improved.

### 2.3. Measures

#### 2.3.1. Neuropsychological Evaluation

The targeted outcome of the intervention was executive function, which is consistent with prior literature on this cognitive domain commonly impacted by exercise and exergaming [9,11,19,22,37,49,58,78,79,103,104]. Executive functioning encompasses higher order cognitive processes, akin to the “CEO of the brain,” planning and directing attention and behavior especially in the face of multiple demands requiring set-shifting, response inhibition, and working memory. These are all key to maintaining independence and avoiding institutionalization in later life [105]. For example, an older adult preparing a meal may need to keep track and manage multiple tasks (e.g., something in the oven and on the stovetop) and, if unsuccessful, red flags may be raised (e.g., smoke alarm triggered or worse). Three measures that tap components of executive function (Stroop, Trails, and Flanker) were administered in electronic form on an iPad via the BrainBaseline software version 2.1 [106].

*Congruent Correct-Incongruent Incorrect Metric (CCII)* [107]. The CCII scaling metric [107] was applied to each of the three executive function measures. The CCII is the percentage of correct congruent responses minus the percentage of the incorrect incongruent responses. This measure is used to gauge the strength of mental processing by quantifying the ability to correctly respond to stimuli when they are relatively easier in contrast to incorrect performance when responding to difficult stimuli. Each of the three executive function measures were scaled to this CCII metric and yielded proportions ranging from −1 to 1 (difference in percentages).

*Stroop.* The Stroop test has long been used to assess executive function in clinical and research samples with good reliability and validity [105,108,109]. The Stroop task evaluates a controlled, effortful response inhibition. In the present study, an electronic version of the Stroop task was administered to participants at each evaluation. An electronic version was administered through an iPad application: BrainBaseline [106].

*Trails.* The original black and white [105,110] and the Color Trails [111] have good reliability and validity and have been used for many years to assess processing speed (connecting the “dots”/circled numbers in order as quickly as possible) and executive function (alternating numbers and a second sequence: letters in the black and white version of Trails B or pink/yellow colored numbers in the Color Trails version). The present study used an electronic form of the Trails task by BrainBaseline [106].

*Flanker.* Additionally, the flanker task was used to further access executive function and stimuli discrimination. The electronic form of the task was used via the BrainBaseline [106] and had participants view five arrows and report which direction the middle arrow is pointing. This task requires dual processing and response inhibition to override the tendency for surrounding arrows to cue a response in a direction different from the actual middle arrow stimuli.

#### 2.3.2. Other Tests Administered

Verbal memory was assessed using the Alzheimer’s Disease Assessment Scale (ADAS) Wordlists for immediate and delayed recall [112,113]. The wordlist task was used to further characterize the sample (e.g., identify participants who might fall into the amnestic subtype of cognitive decline (aMCI) and who might not engage well with the intervention due to a lack of encoding or recall of instructions over time). The ADAS was not hypothesized to change as a result of the iPACES intervention due to memory not consistently being responsive to exercise in prior studies but was included as a manipulation check since the neuro-exergame did have a list-learning task embedded.

The overall cognitive function was assessed with the Montreal Cognitive Assessment (MoCA) and used as a screen for MCI status to characterize the sample. The MoCA is a brief battery of neuropsychological assessments (e.g., clock drawing task, sentence repetition, and letter recognition) that assess various aspects of overall cognitive function. This test has been used frequently over time with high reliably and validity to provide insight of general cognition [114]. The score of MoCA allows for categorization of participant cognitive abilities. Low scores are suggestive of Alzheimer’s type dementia while high scores represent normative cognition and in-between is suggestive of MCI.

*Biomarkers:* Saliva samples were immediately placed on ice until they could be placed in a −80 °C freezer to prevent degradation. Samples were analyzed for concentrations of cortisol (biochemistry lab protocol), DHEA-S (Salimetrics kit), and IGF-1 (Abcam kit). A bicinchoninic acid (BCA) protein concentration from each sample was used to normalize the protein data.

Additional brief questionnaires regarding affective states and experiences while exercising were administered as part of an exploratory addendum study and are reported elsewhere [115].

### 2.4. Materials

**Figure 2:**
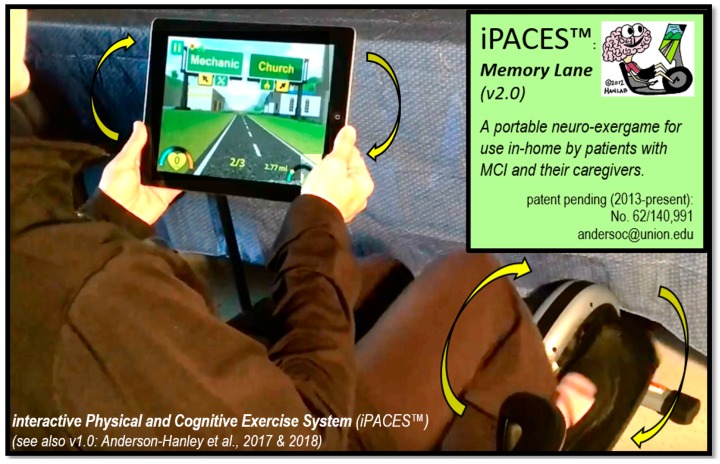
Illustration of the use of interactive pedaling and steering of the iPACES™ v2.0 neuro-exergame, Memory Lane.

The iPACES neuro-exergame studied in this study (described below) was designed by our lab to target a specific neuropsychological function (in this case: executive function given a primary clinical need of the MCI population per above) and was initially deployed on a touch-screen PC tablet (iPACES v1.0) [11,57]. Version 2 (v2.0) of the game utilized in this study, which is now called Memory Lane, was enhanced through a collaboration between our lab and a software company (1st Playable, Troy, NY, USA) to improve graphics, playability, and other features and was deployed in iOS on an iPad 2 Air. Wireless, Bluetooth-enabled devices were integrated to complete the neuro-exergaming interactive operations (e.g., heart rate monitor ring on finger, cadence meter to track the pedaling motion, and an under-desk elliptical pedaler: Stamina 55-1610 InMotion E1000 Elliptical Trainer). Steering along the virtual bike path of the iPACES Memory Lane neuro-exergame was accomplished by holding the iPad like a steering wheel and tilting left and right accordingly to choose a pathway at each fork in the road (Figure 2).

The iPACES Memory Lane game was designed with the premise of challenging and reinforcing executive functions by simulating the naturalistic task of traveling along a roadway to complete a given list of errands (such as: doctor, pharmacy, grocery, starting with 3 and maxing out at 10 locations) and then returning “home,” traversing (and again having to choose the correct errand locations) in reverse order. The game guides the user down a path that leads the user to a fork in the road. The user must steer (tilt) the iPad “left” or “right” to register their choice of errand location per previously given list. If an error is made, the player is given another chance to complete the list of errands in the correct order. Once all the correct locations are chosen, the player encounters a loop in the path that turns them around to choose, in reverse order, the correct forks in the road to the previously completed errand locations.

The iPACES, as played on the iPad, is held like a steering wheel to give the illusion of riding a bike and maneuvering along a scenic path. The iPACES was intended to be used in its full interactive (physical and cognitive exercise) version, but, for experimental purposes, it can also be enabled such that component parts function separately as in “pedaler-only” (physical exercise only) or “game-only” (mental exercise only) each for use as in component familiarization/training and also potentially as comparative control conditions. When in the interactive mode, the speed of the game is controlled by the speed of participant pedaling (picked up through wireless/Bluetooth cadence meter).

*Analyses.* The cognitive outcomes were assessed using paired *t*-tests. Intent-to-treat (ITT) analyses (*n* = 14) were conducted by imputing mean scores for those participants who did not complete the final three-month evaluation (*n* = 7). The relationship between changes in cognition and each biomarker was evaluated with Pearson’s correlations (*r*), which were computed using change scores (post-pre). The significance level was set at *p* = 0.05. Cohen’s effect sizes (*d*) were computed to quantify the magnitude of any significant cognitive effect. For comparison, in lieu of a control group, normative test-retest data was culled from Beglinger’s examination of serial Stroop administration (scaling their reported results by way of converting to percent change with tapering of an initial increase to a plateau, which can be seen as follows: 6%, 5%, 8%, 0%, and 0% and plotted alongside results in this study).

## 3. Results

**Figure 3:**
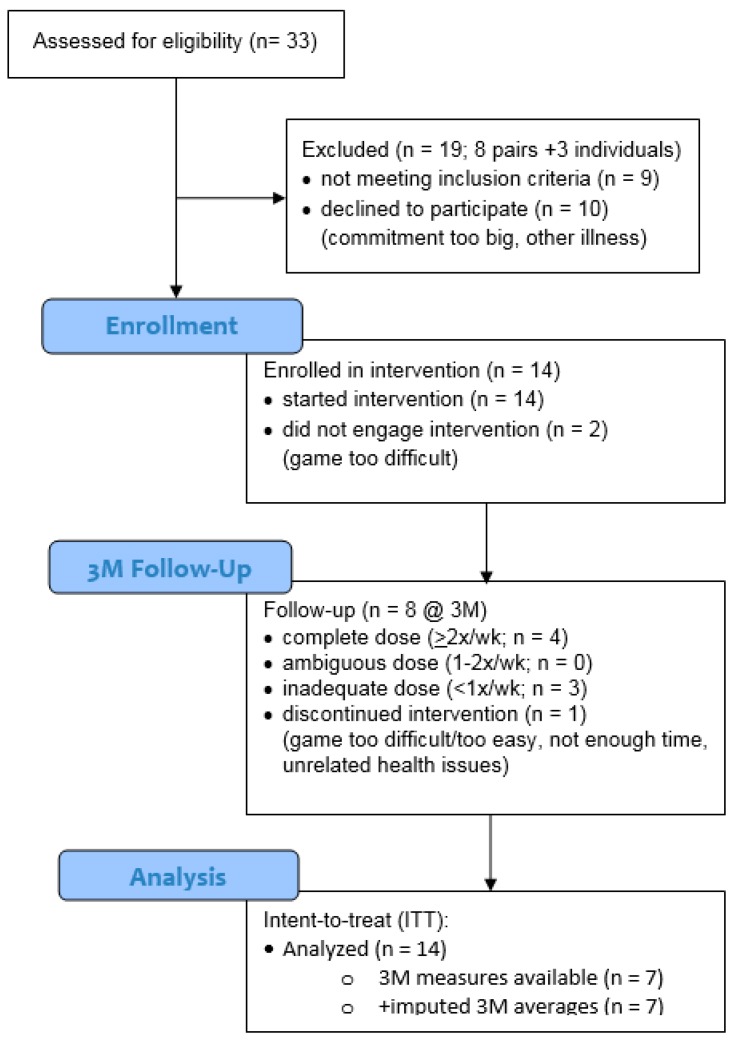
CONSORT Flow Diagram: enrollment and progress of participants through the trial.

Of the 12 participants that were able to provide useable evaluation data, 11 were compliant through the end of the component familiarization period (week 6) and seven were retained through the final three-month evaluation (see Figure 3 CONSORT flow diagram for further details showing participant progress through the trial). No adverse events occurred at any point during the three-month study.

### 3.1. Cognitive Results

Participants’ average cognitive performance across timepoints and conditions is presented in Table 1 including the subvariables that were used to compute the CCII metric for each executive function variable (see above). The three CCII metrics of executive function (Stroop, Trails, and Flanker) were the focus of a priori hypotheses even though only Stroop and Flanker could be analyzed due to too many out-of-range scores on Trails (likely due to multiple restarts from older adult participants whose dexterity or lack of familiarity inflates touch screen sensitivities during required continuous contact while drawing with a finger to connect dots).

**Figure 4:**
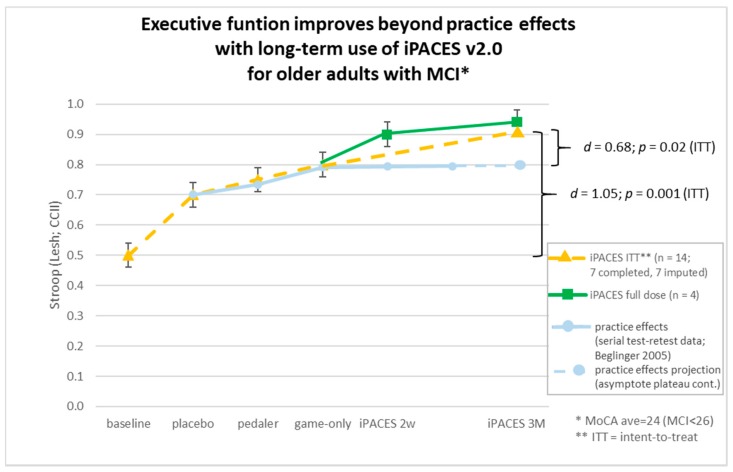
Changes in cognition over three-month iPACES neuro-exergame intervention exceed practice effects.

A significant improvement was found in ITT analyses of executive function (Stroop CCII) from baseline to the three-month iPACES neuro-exergame intervention [*t*(13) = −4.34, *p* < 0.001, *d* = 1.05, Figure 4]. A significant improvement was also observed with a moderate effect size [*t*(13) = −3.53, *p* = 0.004, *d* = 0.68] when comparing as “baseline” the more stringent end of the component familiarization periods (week 6), which was also the start of full interactive iPACES (after which any practice effects were washed out as affirmed by the plotting of the grey line in Figure 4, which shows the plateaued asymptote of Beglinger’s normative test-retest data [101]. See Gray and colleagues for further discussion [116]). Also, plotted for anecdotal visual inspection were the results of those participants that completed the full recommended dose of iPACES (*n* = 4). No significant effect was observed for Flanker.

### 3.2. Biomarker Results

Given the significant improvement in executive function (Stroop) noted above, the correlations between the changes on the three biomarkers (cortisol, DHEA-S, and IGF-1) and the change in Stroop were examined. Greater improvement in Stroop performance was significantly related to a decrease in cortisol (*r* = −0.24, *p* = 0.04) and IGF-1 (*r* = −0.28; *p* = 0.04).

## 4. Discussion

Co-residing pairs of older adults (*n* = 14) were enrolled in a three-month in-home pilot study to examine the cognitive and biomarker outcomes of pedaling and steering an enhanced iPad-based neuro-exergame: the interactive Physical and Cognitive Exercise System (iPACES v2.0). Seven participants (six with MCI) completed the final three-month evaluation and intent-to-treat analyses of all 14 enrollees revealed a significant improvement in executive function (Stroop, but not Flanker) that exceeded practice effects. This was affirmed by evaluating change from the initial baseline as well as from the end of familiarization after washing out serial-testing practice effects and also by comparing with normative test-retest data. The quasi-experimental within-subjects design gradually and sequentially familiarized participants with each of the component conditions (e.g., game-only and exercise-only) before introducing the fully interactive neuro-exergame (iPACES). The effect sizes observed ranged from medium to large even when including those with less than the full recommended dose. As hypothesized, the improvement in executive function was found to significantly correlate with a change in cortisol and IGF-1 (but not DHEA-S). The individuals, which experienced the smallest biomarker changes had the greatest improvement on the Stroop, while individuals with the greatest change in biomarkers had the least Stroop improvement. Contrary to expectations, cortisol significantly increased after the three-month exergame intervention. Although unanticipated, the finding of increased cortisol levels in elderly individuals post intervention has been seen in previous research [117,118].

There are a number of limitations to this pilot study. In particular, the small sample size yields narrow variability of participant limits generalizability, constricts exploration of factors that might affect outcomes, and diminishes power to detect significant effects. Furthermore, the lack of a control group for comparison makes interpretation challenging. However, these results are preliminary given the small sample size and pilot. Due to the quasi-experimental nature of the study, there are a few observations that can be cautiously made to guide further research. First, this pilot study partially replicates and extends our prior pilot of iPACES v1.0 (Anderson-Hanley et al. in press), which confirms that an in-home neuro-exergame intervention for MCI is feasible and potentially effective warranting further study. This is also consistent with other published research on in-home exercise interventions, which are not without their challenges but demonstrate that it is increasingly feasible to incorporate innovated technology into a patient’s home environment [119]. Second, despite a small sample and even with challenges in accruing a full dose of the iPACES intervention, a significant and sizeable effect on one of three measures of executive function was found and the results of this enhanced v2.0 (*d* = 0.68) seem even stronger than the initial pilot of iPACES v1.0 (*d* = 0.39 [11]). Lastly, biomarkers available in readily-obtained saliva samples seem to provide a fruitful avenue for exploring possible underlying mechanisms to explain any cognitive benefits of neuro-exergaming interventions, which is seen with the significant relationship found between cortisol and executive function in other cross-sectional and intervention studies [95,96,97,98,99].

The preliminary pilot findings in this study are consistent with prior research, which has found cognitive benefits of exercise [9] and exergaming [11,56,84]. Chuang and colleagues [120] similarly found an executive function benefit following an exergaming intervention (dance-dance-revolution: DDR). Yet, this pilot adds to the smaller set of literature addressing MCI specifically and extends to prior work on neuro-exergaming by reaching the oft-isolated MCI population with a widely-applicable, safely-seated intervention for in-home use and with an effect size that seems to exceed prior reports [11,56]. There is scant literature on in-home interventions, but notably this echoes Chew and colleagues [120] who reported some benefits to patients enrolled in tandem intervention (physical and cognitive exercises separately). Yet, this study also addresses caregivers directly (some with insidious MCI and/or at least known heavy caregiver burden [121] involving a caregiver in the exercise intervention as directly as participants themselves).

This study had several weaknesses that point to the next steps in the research. The first was a lack of a control group and, while various comparisons were made within subjects (via initial baseline and after washing out practice effects) and also with published normative test-retest data, a matched control group in a larger trial would be more ideal for future research. The small sample does limit statistical power, which means that, while it is encouraging that a significant effect could be detected despite the small sample, a larger sample might shed light on smaller effects that are, perhaps, not detected (e.g., possibly via Flanker) and would also afford greater diversity allowing for more nuanced consideration of generalizability and analytic integration of covariates (such as age, education, sex, etc.). For example, research has shown that benefits of exercise may vary by sex [122,123] and a larger sample could clarify whether there are any inadvertent effects such as ruling out possible gender bias in the choice or applicability of errand locations. Additionally, most participants reported enjoying the study. However, it was often reported that the iPad game became redundant and participants suggested incorporating more challenging and dynamic game features.

The move from paper in the first iPACES pilot to electronic cognitive testing in this second iPACES pilot study revealed some challenges for seniors using the touch screen. For example, high restart and error rates were seen in the Trails task (seven of the 14 participants had >10 restarts on the Trails task at one or more evaluations throughout the study). Additionally, the switch from paper to electronic proved difficult in deriving comparable scores and metrics from a breadth of computer-captured data. The high rate of attrition is not uncommon in intensive exercise interventions [124] and it may have been exacerbated in this study given that these unpaid senior volunteers were either very busy or also dealing with other health and familial complications. Furthermore, it may have been exacerbated in that recruiting pairs was useful for safety afforded via the in-home buddy system but may have also inflated attrition since two were lost when one partner was not able to continue. Last, compliance with the full dose of exercise was challenging for our participants to achieve with four of the seven study completers reaching the recommended target dose of three to five times per week. Participant feedback suggests that those who volunteer for an unpaid study of this sort tend to also be busy with many other commitments, but it may be necessary to further fine-tune the game’s challenge to the ability of the participant. Some participants were only capable of finishing a limited challenge (e.g., maintaining three to four errand locations yet achieving a sense of accomplishment vs. frustration) while others needed a bigger challenge (e.g., maxing out quickly at 10 errand locations and perhaps needing varied scenarios/story-boards to maintain interest such as pedaling along roadways of a state or country to recall and tag a given list of tourist attractions).

Future research might also aim to replicate and extend research on related multimodal interventions [125], which incorporated a nutritional component along with physical and cognitive exercise (in tandem in that study). It makes sense that, if exercise interventions (both physical and mental) are to impact cognitive and brain health via cardiovascular benefits and neuroplasticity, nutritional support for building or repairing neurons could magnify the impact of an intervention. Specifically, an expanding list of nootropics, derived from plant nutraceuticals (e.g., Gingko Biloba, Bacopa Monnieri, Huperzine A, Choline, Phosphatidylserine, Vinpocetine, Rhodiola Rosea, Methylcobalamin) and other potential cognitive enhancers [126] are finding their way into mainstream use among the general population with varying degrees of scientific support on neuronal and brain health [124]. Thus, examining the combined effects of neuro-exergaming with nootropics may be supra-additive in terms of cognitive effects and warrants further study.

Alternative forms of exercise that incorporate interactive physical and cognitive components have been increasingly explored such as dance [127,128,129] and might be contrasted in a future RCT with neuro-exergaming even though care would need to be taken regarding additional variables such as intensity of exercise, aerobic achievement, and influences of socialization. Naturalistic outdoor cycling could be compared with virtual reality cycling and a preliminary pilot in our lab of this type of comparison suggests it is feasible [130], but it is also complex to tease out the impact of multidimensional interactivity, the intensity of cognitive challenges, and the impact of daylight, nature, or green-scapes on outcomes [131]. Examining the role of various forms of mental engagement during exercise could also be fruitful. For instance, this pilot of iPACES asserts a certain level of complexity of the mental challenge (memorizing a list of errand locations and reversing them while pedaling “home”). However, it may also be possible that cognitive performance and brain health is also improved due to less effortful and more relaxing or meditative modes of exercise, which is seen in achieving a flow state while engaging a challenging exercise or in Tai Chi [132].

Additional biomarkers might also be useful to examine in future research such as the brain-derived neurotrophic factor (BDNF), which has been found to predict a cognitive benefit of a dual-task paradigm for those with MCI [133].

## 5. Conclusions

In conclusion, the results of this pilot study indicate that a portable, iPad-based neuro-exergame is feasible for MCI and caregiver co-residing pairs to use in the home and it appears to have a sizeable effect on executive function, which warrants further research. It is anticipated that there will continue to be calls for additional research and funding for RCTs to evaluate these types of innovations for addressing the encroaching dementia epidemic [75,134]. It is hoped that these interventions could potentially ameliorate the cognitive decline of increasing numbers of those with MCI [75] including reaching them at home where they often become secluded. Perhaps with enough evidence, the scientific community might arrive at “prescribable video games” as a non-pharmacological intervention to address cognitive decline [135] or, as in this pilot study of iPACES (v2), an impactful multimodal neuro-exergame intervention.

## 6. Patents

iPACES™ patent pending (US15087351).

## Figures and Tables

**Table 1:** Cognitive and biomarker data. Note: **bold** indicates *p* < .05 (ITT: *n* = 7, and ITT imputed: *n* = 14) compared both with baseline and end of component familiarization (end of game-only).

		0		2w		4w		6w		8w		3M	
		baseline (*n* = 14)		placebo (*n* = 12)		pedaler (*n* = 11)		game (*n* = 11)		iPACES (*n* = 11, 7)			
		ave	SD	ave	SD	ave	SD	ave	SD	ave	SD	ave	SD
*Stroop*	Incongruent % correct	0.61	0.31	0.77	0.33	0.78	0.28	0.86	0.13	0.79	0.24	0.93	0.05
	Congruent % correct	0.88	0.13	0.94	0.10	0.97	0.06	0.94	0.07	0.96	0.05	0.98	0.03
	Incongruent ave time (s)	1.45	0.22	1.38	0.17	1.42	0.22	1.41	0.21	1.35	0.16	1.33	0.16
	Congruent ave time (s)	1.37	0.18	1.22	0.25	1.13	0.14	1.22	0.17	1.23	0.19	1.10	0.07
	Total duration (s)	269.0	104.3	186.8	63.7	149.9	68.3	107.3	54.9	100.6	44.6	70.7	5.7
	CCII	0.48	0.41	0.71	0.39	0.75	0.33	0.80	0.16	0.75	0.28	**0.91**	0.07
*Trails*	restarts	4.77	6.56	2.00	1.95	1.64	2.25	1.36	1.12	0.73	1.19	1.43	1.90
	A % error	0.42	0.58	0.20	0.24	0.15	0.23	0.11	0.09	0.06	0.09	0.13	0.16
	B % error	0.29	0.27	0.18	0.17	0.16	0.16	0.10	0.10	0.10	0.09	0.28	0.15
	B duration (s)	212.6	175.5	160.9	151.6	145.8	81.1	124.5	69.2	125.0	81.0	163.1	55.4
	CCII	0.28	0.73	0.64	0.31	0.69	0.30	0.79	0.16	0.84	0.15	0.59	0.28
*Flanker*	Incongruent % correct	0.81	0.25	0.91	0.14	0.90	0.17	0.93	0.07	0.92	0.12	0.76	0.36
	Congruent % correct	0.91	0.10	0.89	0.14	0.97	0.03	0.95	0.08	0.95	0.08	0.98	0.02
	Incongruent ave time (s)	0.78	0.21	0.65	0.08	0.71	0.12	0.66	0.12	0.64	0.06	0.77	0.22
	Congruent ave time (s)	0.74	0.25	0.63	0.08	0.64	0.07	0.61	0.12	0.61	0.07	0.67	0.12
	Total duration (s)	244.9	47.7	207.9	94.2	152.7	63.4	97.8	64.5	97.4	57.9	71.9	16.7
	CCII	0.72	0.28	0.79	0.27	0.87	0.19	0.87	0.13	0.87	0.20	0.74	0.36
*ADAS*	Word List (sum trials correct)	18.64	4.58	19.29	5.68	20.33	4.33	19.92	3.96	21.00	4.63	21.13	4.67
	Word List (delay correct)	5.08	2.66	5.85	2.44	5.58	2.23	6.42	2.02	5.75	2.83	5.63	2.13
*Biomarkers*	cortisol	3.55	3.36	5.08	5.37	5.52	5.23	2.58	3.38	3.06	4.00	17.61	7.17
	DHEA-S	8733	5923	7729	8620	7301	9153	6508	6085	4999	3547	6072	6503
	IGF-1	3.16	2.73	3.43	3.66	1.82	1.01	2.64	1.91	1.83	1.37	2.56	2.46

Notes: **bold** indicates *p* ≤ 0.05 (ITT: *n* = 7, and ITT imputed *n* = 14) compared both with baseline and end of component familiarization (end of game-only).
